# Denosumab combined with precision radiotherapy for recurrent giant cell tumor of the thoracic spine: a case report and literature review

**DOI:** 10.3389/fneur.2023.1308600

**Published:** 2024-01-04

**Authors:** Zukang Miao, Ming Xu, Kai Zheng, Hai Gong, Ning Yan, Qian Chen, Xiuchun Yu

**Affiliations:** ^1^Department of Orthopedics, The 960th Hospital of the PLA Joint Logistics Support Force, Jinan, China; ^2^First Clinical Medical College, Shandong University of Traditional Chinese Medicine, Jinan, China; ^3^Department of Radiotherapy, The 960th Hospital of the PLA Joint Logistics Support Force, Jinan, China

**Keywords:** giant cell tumor of bone, spine, local recurrence, radiotherapy, denosumab

## Abstract

Giant cell tumors of the spine have a high recurrence rate owing to their special anatomical site; hence, further treatment after recurrence is very challenging. Achieving effective tumor control and improving the long-term quality of life of the patients are the main treatment purposes to consider for recurrent giant cell tumors of the spine. A patient showing giant cell tumor recurrence of the thoracic spine after curettage received denosumab combined with precision radiotherapy, through which the tumor gained good control and the patient could regain normal functioning. A review of the relevant literature suggested that denosumab combined with radiotherapy is an effective new approach for the treatment of recurrent giant cell tumors of the spine.

## 1 Introduction

Giant cell tumor (GCT) of the bone is an osteolytic, aggressive primary bone tumor that can manifest in the epiphysis of the limbs, sacrum, spine, and other places, mostly in adults aged 20–45 years (1). GCT comprises mononuclear stromal cells and characteristic multinucleated giant cells exhibiting osteoclastic activity that can modify the appearance of normal bone swelling and the destruction of the bone cortex (2). Although the incidence of spinal GCT is low, accounting for only 3% of all GCT cases (3), the tumor tissues surrounding the spinal cord and nerve roots are not easily accessible owing to their physiological and anatomical structure. It significantly increases the difficulty of extensive resection because it requires the resection of the margin or the inner edge of the lesion, which further contributes to its higher recurrence rate. Long-term follow-up has indicated that surgical treatment alone is associated with a local recurrence of the tumor in 15–50% of the patients (4). For recurrent spinal GCT after surgery, reoperation can result in extensive surgical trauma and functional damage, which is an important factor to consider when selecting the treatment modality. We have hereby presented the report of a patient with recurrent thoracic GCT in our hospital, in whom denosumab combined with precision radiotherapy achieved effective tumor control. Accordingly, based on our successful experience, we have proposed a new treatment concept, reviewed the recent relevant literature on recurrent spinal GCT treatment, and summarized the latest treatment strategies to provide a reference for adaptation in clinical practice.

## 2 Case report

**Figure 1:**
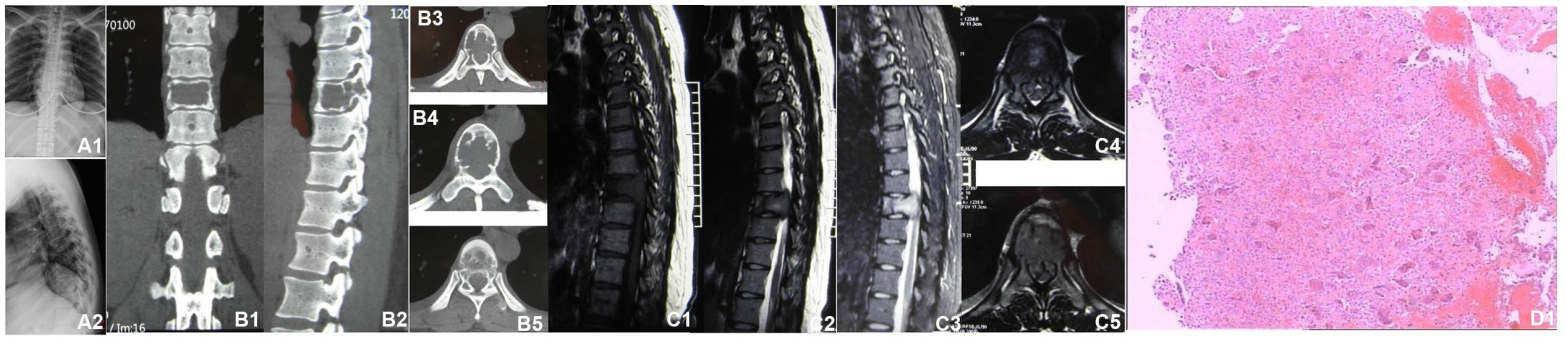
Imaging data at the initial clinic visit (2020-04). **(A1,A2)** Thoracic vertebral X-ray: The local density of the T9 vertebrae was decreased. **(B1–B5)** Thoracic vertebral CT: T9 vertebral bone destruction, cortical destruction at the upper, lower, and posterior margins, bilateral bone destruction of the pedicle, an irregular soft tissue density shadow visible in the vertebral body, and the spinal canal were occupied, with a corresponding spinal canal sagittal diameter narrowing. **(C1–C5)** Thoracic vertebra MRI: T9 vertebral body flattening, and long T1 and long T2 signals appearing within the vertebral body. The fat suppression phase revealed a high signal intensity, bilateral pedicle involvement, bone destruction at the posterior edge of the vertebral body, soft tissue space, and dural compression. **(D1)** Puncture pathology revealed massive osteoclasts, considering the giant cell tumor of the bone.

**Figure 2:**
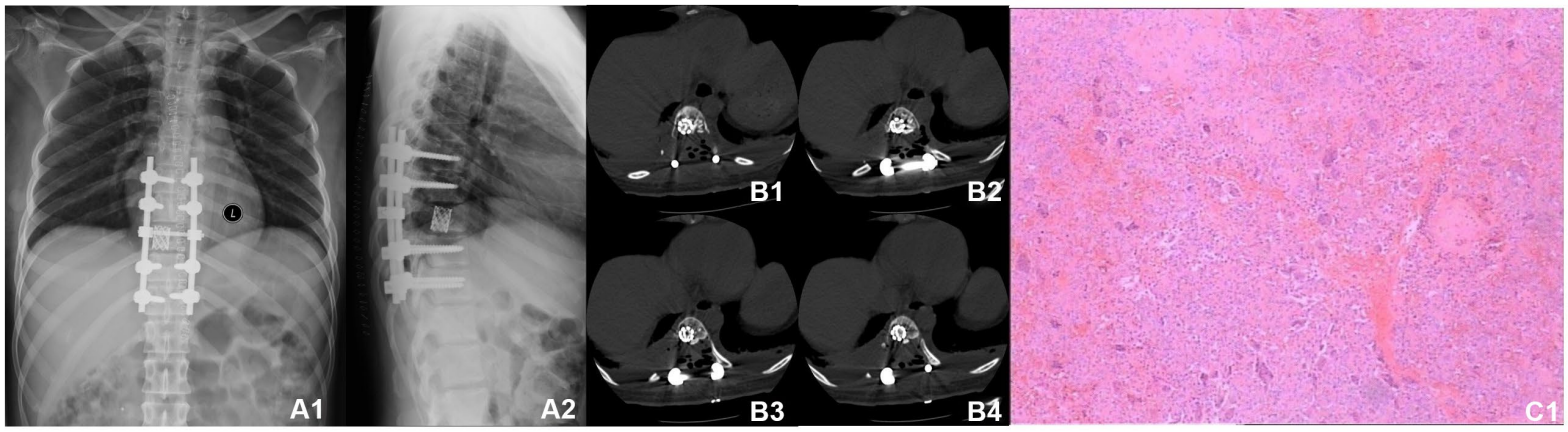
Postoperative radiographic data (2020-04). **(A1,A2)** Thoracic vertebral X-ray revealed a satisfactory internal fixation position; the placement was visible in the T9 vertebra. **(B1–B4)** Thoracic vertebral CT showing T9 vertebrae filled with a high-density shadow. **(C1)** Postoperative pathology revealed a T9 vertebral giant cell tumor of the bone.

**Figure 3:**
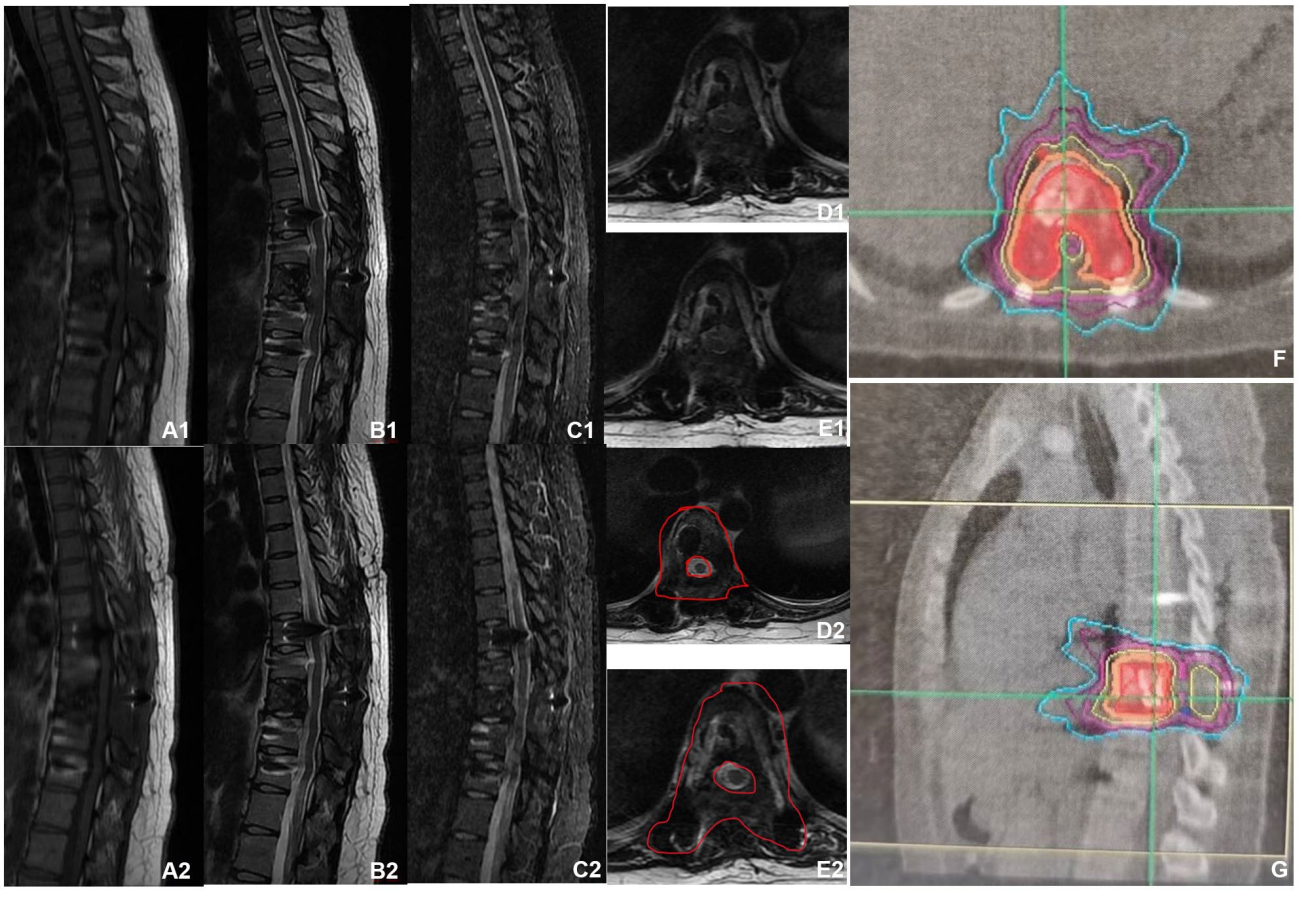
MRI before and after denosumab administration. **(A1–E1)** MRI before denosumab administration showed uneven bone signals in the T9 vertebrae, with patchy abnormal signals at the posterior margin of the vertebral body. Burst into the spinal canal, and spinal cord compression can be seen. **(A2–E2)** MRI of the thoracic spine after three doses of denosumab administration (2021-02). The space in the spinal canal is significantly smaller than that at the front, and the boundary with the spinal cord is clear. **(F,G)** Preoperative planning for cyber knife radiation therapy.

**Figure 4:**
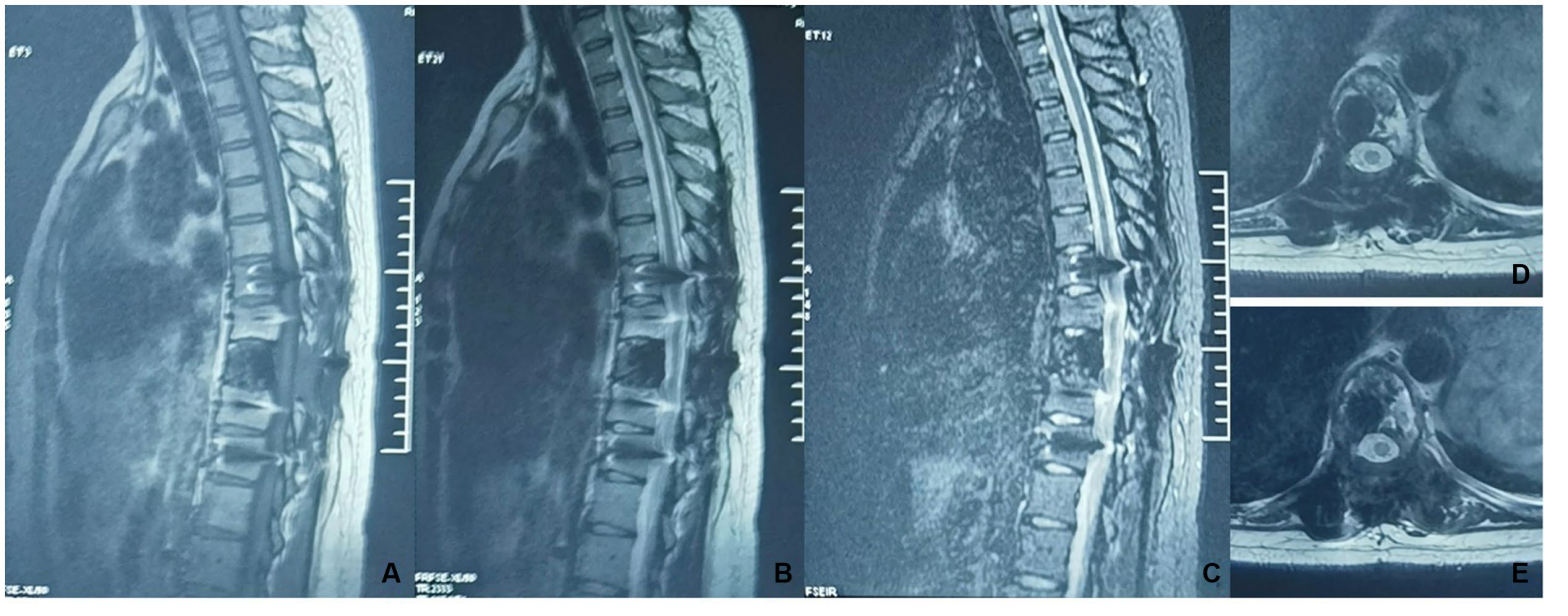
A thoracic MRI was reviewed after radiotherapy (2023-10). **(A–E)** Non-uniform hypointensity was observed in the T9 vertebrae, with no significant abnormalities in the intraspinal spinal cord.

A 30-year-old woman without a family inherited disease presented to our hospital on 14 April 2020 for the “numbness of both lower extremities since 11 days after a fall.” Her physical examination revealed no pressing pain in the chest or upper back. She experienced hypoesthesia below the navel, on bilateral thighs, the calf front, and the back of the foot skin, especially on the left side. The muscle strength and muscle tension of both lower limbs were found to be normal. The remaining physical examination revealed no evident abnormalities. Thoracic spine X-ray and computed tomography (CT) demonstrated abnormal bone destruction in the T9 vertebral body. Thoracic magnetic resonance imaging (MRI) displayed abnormal signal changes in the T9 vertebral body and space-occupying lesions in the spinal canal. CT-guided downward T9 vertebral tumor puncture biopsy and consideration of the puncture pathology indicated a GCT of the bone (Figures 1A–D). The parents are in good health and have no underlying diseases. After excluding surgical contraindications on 17 April 2020 and after internal fixation of the posterior T9 vertebral tumor microwave with an inactivated curettage graft, the postoperative pathology was the same as puncture pathology (the tumor of the T9 vertebral body was scraped after the microwave, and the titanium cage filled with autogenous bone was supported in the scraped vertebral body). T7, T8, T10, T11 bilateral screws were fixed. After the operation, the patient reported no numbness in either lower limb, a well-healed incision, and no other discomfort. Accordingly, the patient was discharged after removing the stitches (Figures 2A–C). To prevent tumor recurrence, zoledronic acid (4 mg) was administered once a month after the surgery. She was admitted for the eighth sequential postoperative zoledronic acid treatment on 26 January 2021, and no evident abnormalities were detected during her physical examination. A review of the thoracic spine MRI revealed that the spinal cord compression had an irregular signal in the spinal canal. The patient’s past medical history and the postoperative recurrence of GCT were considered. After three treatments with denosumab (120 mg), the thoracic spine MRI was reviewed again. The tumor boundary in the vertebral body was clear, with no invasion of the spinal cord. After consultation in the radiotherapy department, stereotactic radiotherapy was performed for recurrent lesions (cyber knife) with 600 cGy/fraction*6 fractions, 1 fraction/day (Figures 3A–G). Tumor control was achieved after the last radiotherapy, with no evident abnormalities detected. At the latest follow-up in October 2023, no tumor progression was found (Figures 4A–E). The patient has returned to normal life without significant complaints.

## 3 Literature review

### 3.1 Criteria for literature selection

Inclusion criteria: (1) GCT of the bone was diagnosed; (2) GCT development in the spine and recurrence after initial treatment; (3) systematic diagnosis after recurrence and treatment was performed, and the prognosis was clearly observed; (4) retrospective analyses or individual case reports.

Exclusion criteria: (1) case reports without a systematic diagnosis or treatment process; (2) reports of malignant tumor changes before recurrence; (3) literature review and meta-analysis; (4) literature of repeatedly reported cases.

### 3.2 Literature search strategy

The search terms “giant cell tumor of bone,” “spine or spinal,” and “recurrent or recurrences” were used for literature published in PubMed and the Web of Science from 2010 to 2022 to search for relevant studies.

### 3.3 Literature search results

After screening by applying the inclusion and exclusion criteria, a total of 9 articles were shortlisted (Table 1) (5–13), which included 7 case reports and 2 case series reports, totaling 21 patients (7 male patients, 14 female patients; age: 11–64 years); presenting with cervical vertebrae (*n* = 2); thoracic vertebrae (*n* = 10); lumbar vertebrae (*n* = 7); sacral vertebrae (*n* = 2); treatment modalities included sodium ibandronate, radiotherapy, surgical resection, chemotherapy, interferon administration, and denosumab administration.

**Table 1:** Cases in the literature.

No.	Year	Study	*N*	Location	Age and sex	Treatment	Outcome
1	Zhang et al. (5)	Case series	3	T7 /L5/ S1 and S2 vertebrae	23/32/33-year-old woman	Sodium ibandronate	The studies reveal potential promise for the use of sodium ibandronate to treat recurrent GCT. Moreover, it is required to verify the safety and effectiveness
2	Meyer et al. (6)	Case report	1	T7 vertebral	64-year-old female	Radiotherapy	Radiation therapy remains an appropriate therapy option in benign giant cell tumors with minimal adverse sequelae if primary surgical treatment is not feasible or fails
3	Agarwal et al. (7)	Case report	1	T6 vertebral	27-year-old woman	Denosumab and Surgical resection	Denosumab treatment markedly shrank the tumor and enabled complete surgical resection
4	Luo et al. (8)	Case report	1	L4 vertebral	11-year-old boy	Surgical resection and Denosumab	Surgical resection is the first choice Denosumab should be utilized after tumor resection whether based on the purpose of prevention or treatment of tumor recurrence
5	Duan et al. (9)	Case report	1	T11, T12 vertebrae	50-year-old woman	Denosumab and Total en bloc spondylectomy	Denosumab therapy contributes to tumor regression. TES may be an effective and feasible strategy for managing huge recurrent GCTs of the spine after denosumab therapy
6	Shirzadi et al. (10)	Case report	1	C2 odontoid process	15-year-old boy	Surgical resection, Radiation, Proton beam therapy, Chemotherapy and resection	An aggressive surgical approach with the goal of complete resection, adjuvant treatment with chemotherapy and radiation therapy, and long-term frequent follow-ups for recurrence should be considered the optimal treatment
7	Wei et al. (11)	Case report	2	C1-2/T5-6	29-year-old woman/ 21-year-old man	Interferon alfa-2b (IFNɑ-2a)	Interferon therapy may be an effective and safe option for recurrent giant cell tumors in spine
8	Lin et al. (12)	Case series	10	5 spinal GCTB were located in the thoracic spine, 4 in the lumbar spine, and 1 in the sacrum	28.9 (range 21–40 years), 3 men and 7 women	1 patients: Surgeries; 3 patients: Surgeries+Bis-phosphonates; 1 patients: Surgeries+ Bis-phosphonates+Denosumab; 1 patient: Denosumab; 1patient:Radiotherapy+Zoledronate+Denosumab; 3 patients: Bisphosphonates	Intralesional excision for recurrent spinal giant cell tumors is an effective option that may have a satisfactory prognosis; Adjuvant treatments perioperatively and systemic medical treatments can have therapeutic effects in the recurrent SGCT
9	Guo et al. (13)	Case report	1	L2 vertebrae	51-year-old man	Denosumab and Total en bloc spondylectomy for GCT reconstructed using 3D-printed vertebrae	Multilevel lumbar TES for GCT reconstructed using a 3D-printed vertebrae is an effective option for curative management of GCTs

## 4 Discussion

Spinal GCT can involve the adjacent vertebrae through adjacent joints (14). When the tumor expands into the spinal canal, the spinal cord and the associated nerve roots and blood vessels often get compressed, resulting in a free degree of lower back pain or even paraplegia (15). Generally, curettage or partial or total vertebral resection is selected for spinal GCT based on the Enneking staging of the tumor (16). Previous studies have reported postoperative local recurrence rates of 20–50% with spinal GCT, with the maximum rate reaching 70% due to the difficulties encountered in complete resection (17, 18). The recurrence rate of local tumors is closely correlated with the site of the tumor and the degree of primary surgical intervention (6). Curettage may cause minor tissue damage but a relatively high local recurrence rate. Resection poses a lower risk of local recurrence but can result in relatively severe tissue damage and serious complications (19, 20). It is important to consider that the primary therapeutic goal of GCT is to provide long-term symptom relief, especially from pain, as well as tumor control to maintain the long-term good functional status of the patient (21). Therefore, seeking an approach with a low recurrence rate and good functional retention is an important choice in surgery.

Cervical spinal tumors, curettage, intralesional curettage, and non-intact tumors are the risk factors associated with local recurrence (22). The use of adjuvant therapy during and after surgery can reduce the risk of recurrence of GCTs from 45–65% to 12–18% (23). For example, the application of high-speed burring facilitates the selection of tumor curettage and ensures the adequacy and quality of curettage (24). According to the literature, tumor inactivation was performed using frozen, phenol, alcohol, and phenol–alcohol combinations. The scraped cavity was filled with poly(methyl methacrylate) (PMMA) and acrylic cement, and the heat released by the polymerization was applied to induce tissue necrosis, and the resultant cytotoxicity was used to create hypoxia in the cells. Long-term postoperative use of bisphosphonates (25–28) is believed to significantly reduce the local recurrence rate while preserving the neurological functions of the patient (29). Yu et al. (30) and Zheng et al. (31) also indicated that the use of cementation after curettage shows promise in limiting early postoperative complications, lower recurrence, and easier usage in general.

Regular re-examination is critical to detecting tumor recurrence over time. Asymptomatic recurrent spinal GCT is uncommon. The lower back pain and neurological dysfunction of recurrent spinal GCTs are mostly caused by advanced lesions with intraspinal tumor spread. A recent study (32) showed that in spinal tumors, the most common cause of revision was tumor progression (66.7%). More aggressive surgeries (en bloc or gross total) are considered the best option for the treatment of a recurrent primary tumor (33). The feasibility and applicability of reoperation for recurrent spinal GCT are extremely limited; it is inoperable owing to the location of the tumor, and secondary surgery can result in unacceptable functional defects. Even an apparently appropriate en bloc resection can be unsuccessful (34). As the literature points out (1, 5, 6, 12, 13), direct reoperation alone is the only way to remove recurrent tumors. En bloc resection requires sacrificing not only the affected bone but also almost all connecting elements, creating full instability (35). A contemporary series of GCTs in the spine reported a perioperative death after neurologic decline postoperatively, which highlights the risks involved with these surgeries (36). Therefore, non-surgical treatment or combination therapy may be considered a better alternative.

Denosumab has been formally applied in the treatment of patients with unresectable GCT of the bone, indicating promising efficacy and biological integrity. It controls the progression of GCT by inhibiting osteoclast-mediated bone destruction and reducing the tumor blood supply (37). Denosumab is a fully human monoclonal antibody to the receptor activator of the nuclear factor kappa B ligand (RANKL). Presently, preoperative denosumab is not recommended as it can result in local bone sclerosis, unclear tumor boundaries, and insufficient curettage of tumors, thereby contributing to a high tumor recurrence rate. However, it has achieved important efficacy for recurrent or inoperable GCT (38–40), which can significantly reduce the tumor size and protect the integrity of the adjacent bone tissues. Boriani et al. (41) also demonstrated that denosumab can be considered an excellent solution in spine GCTs whose surgical treatment cannot be Enneking appropriate or is associated with unacceptable morbidity or loss of function. There is evidence that the discontinuation of the treatment can be associated with tumor progression. Because it is still unclear at what minimum effective dose and time interval this drug can be safely injected, it is still impossible to state when to safely stop the treatment (42, 43). As Luo et al. (8) said, an 11-year-old patient achieved tumor control but was unable to stop denosumab. Therefore, denosumab is a more beneficial and rational application that deserves further consideration by our clinicians.

GCTs are highly sensitive to radiotherapy, and local radiotherapy has demonstrated good outcomes in long-term local tumor control and the incidence of adverse events (44). Previous studies have reported serious complications from reoperation, such as resident tumors from surgical margin incision or recurrence; hence, radiotherapy should be considered, which has been associated with controllable postoperative complications (45). In addition, the response rate of radiotherapy is 100%, with an overall survival rate of 98% and an overall local control rate of 79% (44). A recurrent tumor is an indication for radiotherapy (46). According to past studies, radiotherapy at a dose of 40–45 GY is highly effective, although better outcomes have been achieved with a total dose of GCT >45 GY while considering the special anatomy of the spinal cord. Considering the specific anatomical structure of the spinal cord, no local control rate was found to improve despite increasing the total radiation dose (47, 48). However, the local benefits of radiotherapy are debatable, and the risk of secondary malignancy cannot be excluded (49, 50). Nevertheless, with the advancements in radiotherapy technology, such as the development of 3-dimensional conformal radiotherapy and intensity-modulated radiotherapy, an adequate radiation dose can be produced with lower radiation toxicity, and the key anatomical structures and important tissues can be safeguarded. When the tumor cannot be completely excised or subjected to curettage in patients presenting with multiple recurrences, radiation therapy can be considered to achieve effective control of the tumor. However, when recurrent tumors have invaded the neurospinal cord, the use of radiotherapy is limited. As reported in the literature (6), even if tumor control is achieved, paralysis of the patient cannot be avoided.

Selective arterial embolization (SAE) is also an effective approach to reducing or ossifying the tumor, which can alleviate pain, stabilize lesions, and improve survival in the presence of adequate blood supply to spinal GCT (51). N-butyl 2-cyanoacrylate (NBCA), as a new embolic agent for preoperative endovascular embolization and vascular embolization of recurrent cervical GCT, can not only significantly reduce postoperative bleeding but also reduce the chance of recurrence (52). Literature reports the application of doxycycline sclerotherapy in the treatment of axial skeleton cases of postoperative recurrence and the inability to undergo surgery (53). Interferon alfa-2b (IFNɑ-2a) achieves good tumor control via its anti-tumor and angiogenic effects (11). There is a lack of reports with a high level of evidence.

In the present case, after curettage of thoracic GCT, the continuous application of bisphosphonates continued to reduce the chance of recurrence. After 9 months of the operation, the tumor recurred. Although the patient did not complain of any obvious discomfort and showed no positive signs after physical examination, the imaging indicated the invasion of the recurrent tumor into the spinal canal and the adjacent running nerve. This event highlights the need to conduct a timely intervention to avoid further tumor progression and serious complications. Although the efficacy of radiotherapy and denosumab for recurrent GCT has been fully verified, the recurrent tumor in this patient has invaded the spinal canal and nerve. Thus, it is evident that the blind use of radiotherapy can damage the important tissues surrounding the tumor. Therefore, after multidisciplinary consultation and discussion, denosumab was used first in the present case, which significantly narrowed the tumor and showed clear boundaries with the surrounding dural and nerves. Accordingly, local radiotherapy was performed six times to achieve good tumor control. There has no tumor progression in the 33 months follow-up.

## 5 Conclusion

The successful application of denosumab combined with radiotherapy implies that this new treatment modality can be applied to relapsed spinal GCT in order to achieve maximum control of tumors with minimal damage. Therefore, a combination of multiple methods is deemed optimal to achieve better outcomes. We believe that the proposed therapeutic approach can serve as a reference for future development and application. For recurrent GCT in spine, radiotherapy may be useful in order to avoid denosumab dependence.

## Data availability statement

The original contributions presented in the study are included in the article/supplementary material, further inquiries can be directed to the corresponding author.

## Ethics statement

The studies involving humans were approved by the 960th Hospital of the PLA Joint Logistics Support Force. The studies were conducted in accordance with the local legislation and institutional requirements. The participants provided their written informed consent to participate in this study. Written informed consent was obtained from the individual(s) for the publication of any potentially identifiable images or data included in this article.

## Author contributions

ZM: Data curation, Investigation, Resources, Writing – original draft. MX: Investigation, Resources, Supervision, Writing – review & editing. KZ: Investigation, Resources, Supervision, Writing – review & editing. HG: Investigation, Resources, Writing – review & editing. NY: Investigation, Resources, Writing – review & editing. QC: Investigation, Resources, Writing – review & editing. XY: Resources, Supervision, Validation, Writing – review & editing.
